# La Grippe—Three Cases

**Published:** 1890-02

**Authors:** H. B. Stiles

**Affiliations:** Abilene, Texas


### LA GRIPPE—THREE CASES.

H. B. Stiles, A. M., M. D., Abilene, Texas.

We are in the midst of an epidemic of la grippe.

In response to your request for reports of treatment, I forward accounts of three cases, typical of my three chief remedies.

Case I.—C. J., æt. thirteen. Attack began with severe aching of head and frame throughout. Headache frontal, heavy, bursting. Eyes would feel as if falling out. Tongue white. Patient thirsty for frequent small drinks. Very bad, putrid taste in mouth. Patient restless—tossing and rolling. Fever 104.5°.

℞ Baptisia$^{cm}$. Briefly, the case improved forthwith, and all febrile symptoms, aching, and restlessness were cured with Baptisia. The persisting spasmodic cough, worse at night and worse lying down, was cured with Drosera.

Case II.—Mr. J. Father of above patient.

Symptoms: Fever 104.5°. Headache very severe, with splitting sensation when coughing. Pains in back, shoulders, loins—in fact, all over, $<$ by motion—℞ Baptisia, which seemed to be *genus epidemicus*. It lowered fever some, but that was all.

Later symptoms: Sharp pains in pleura when coughing. Rusty sputa. ℞ Bryonia, which at once reduced fever almost completely, relieving headache and other aches at same time, soon curing the pneumonia also.

Case III.—Miss Q. Brunette.

While at duty in school on Friday morning was seized with very severe aching of back, neck, and head.

Coming to office at noon received Bapt.$^{cm}$.

Calling to see her next morning I was informed that during the preceding afternoon she had suffered extremely with headache, of a bursting, throbbing character. Back, neck, and limbs all joining chorus of aching, head leading.

Fever rose very high, probably 104°. Fever was then (Saturday morning) evidently absent, though, having left my thermometer, I cannot affirm that it was. Still, her head ached intolerably. Eyes sensitive to light, pupils dilated. Said that on previous afternoon she "almost went wild" from nervousness, caused by listening to talk of visitors. Every sound aggravated. Nervous as lightning.

Now, while this case resembled Case I in the aching, thirst, and restlessness, in general, it yet differed in having a greater degree of erethism—patient was more excited and excitable. All her symptoms were more active and poignant than in Case I.

℞ Belladonna every two hours, and forbade company. Saturday evening all pains and aches were relieved. Headache came in paroxysms instead of being continuous. Continued Belladonna. Sunday evening, temperature 99°. All pains gone. Patient discharged.

These three cases are typical of the Abilene epidemic.

The Bryonia case is easily recognized by its agg. by motion.

The Bell. case is not so easily distinguished from the Bapt. case, but has, as said above, a greater activity, a more vigorous nervousness, with severe neuralgic pains.

The first part of the Bapt. case is likely to show extreme nervousness, but its tendency is to become more and more quiet—to take the *low* appearance.

Drosera and Pulsatilla have served me best in the cough—prescribed according to symptoms.

We Southern physicians recognize in this world-wide grippe our old enemy *the dengue.* It has the aching; it has the putrid taste; it has the debility and persistence of neuralgia after convalescence that marked the epidemic of dengue in 1885. Some few cases have the eruption also which marked many cases of dengue.

Owing, I believe, to the season, it has more of the catarrhal and less of the "bilious" character of former invasions of dengue, though in some cases that also resulted in pneumonia.

I have found Bapt. superior to Aconite as antipyretic in these cases, and superior to Eupatorium for relieving the aching. It is the most frequently used remedy.

